# Prevalence and risk factors of vitamin D deficiency in inherited ichthyosis: A French prospective observational study performed in a reference center

**DOI:** 10.1186/s13023-014-0127-3

**Published:** 2014-08-05

**Authors:** Flora Frascari, Isabelle Dreyfus, Lauriane Rodriguez, Isabelle Gennero, Khaled Ezzedine, Jean-Pierre Salles, Juliette Mazereeuw-Hautier

**Affiliations:** 1 Paul Sabatier University and Department of Dermatology, Rare Skin Diseases Reference Center, Larrey Hospital, CHU Toulouse, Toulouse, France; 2 Molecular Signaling of Growth, Osteogenesis and Osteolysis, Biotherapy, INSERM UMR 1043, CNRS U5282, Université Paul Sabatier, Centre de Physiopathologie de Toulouse Purpan (CPTP), Toulouse, France; 3 Biochemistry Laboratory, Federative Institute of Biology, Toulouse University Hospital, Toulouse, France; 4 Department of Dermatology and Pediatric Dermatology, National Center for Rare Skin Disorders, Saint André Hospital, Bordeaux University Hospital, Bordeaux, France; 5 Unit of Endocrinology, Genetics, Gynecology and Bone Diseases, Children’s Hospital, Toulouse, National Center for Rare Diseases of Calcium and Phosphorus metabolism, Toulouse, France

**Keywords:** Inherited ichthyosis, Genodermatosis, Vitamin D deficiency, Prevalence, Risk factors

## Abstract

**Background:**

To date, few studies have investigated serum vitamin D status in patients with inherited ichthyosis. The aim of this study was to determine the prevalence of vitamin D deficiency (defined as serum level <10 ng/mL) in a French cohort of patients and to identify associated risk factors.

**Methods:**

This was a prospective observational study performed in a hospital reference center with expertise for rare skin diseases. Patients’ clinical characteristics were recorded. Serum concentration of 25-hydroxyvitamin D and parathyroid hormone were determined. For patients with vitamin D deficiency, serum calcium, serum phosphorus and bone mineral density were also investigated. Comparisons between groups (25-hydroxyvitamin D <10 ng/mL versus ≥10 ng/mL) were conducted by univariate and multivariate logistic regression.

**Results:**

Of the 53 included patients, 47 (88.7%) had serum 25-hydroxyvitamin D below the optimal level of 30 ng/mL: 18 (34%) had vitamin D sufficiency, 14 (26.4%) had vitamin D insufficiency, and 15 (28.3%) had vitamin D deficiency. A negative linear correlation was found between 25-hydroxyvitamin D and parathyroid hormone levels for the whole study population. Serum calcium and phosphorus levels were normal for the 15 patients with vitamin D deficiency. Bone mineral density was investigated for 11 of these latter 15 patients, and six of them had osteopenia. Winter/spring seasons of vitamin D measurement, severity of ichthyosis, and phototypes IV–VI were identified as independent risk factors for vitamin D deficiency.

**Conclusions:**

Clinicians should be aware of the risk of vitamin D deficiency in the management of patients with inherited ichthyosis, especially in winter and spring, and in case of dark skin or severe disease.

## Background

Inherited ichthyoses are monogenic disorders of cornification due to mutations in genes involved in skin barrier function. Skin changes involve most of the tegument, and are characterized by scales of various forms and severities, and these are often associated with erythema. According to a recent ichthyosis expert consensus, the classification should be clinically based, and distinguishes syndromic from nonsyndromic ichthyoses [1]. No curative treatment is available; however, skin condition is usually improved by emollients, and acitretin may be used for severe cases. Quality of life is usually affected, in particular because of troublesome symptoms such as cutaneous pain and pruritus [2],[3]. Ichthyoses may be complicated by episodes of skin infection, heat intolerance, and deafness due to accumulation of scales in the ear canal. Abnormally low vitamin D (vitD) levels have also been reported as a possible complication of the disease [4]–[13].

The role of vitD in bone mineralization is well documented [14], but it is also thought to have an effect on cancer and immune disorders [15],[16]. The main source of vitD occurs when skin is exposed to UV B from sunlight, when provitamin D is converted into previtamin D. VitD may also be provided by food sources. Whatever its origin, vitD requires enzymatic conversion in the liver followed by enzymatic conversion in the kidneys to become active [vitD is converted to 25-hydroxyvitamin D (25-(OH) D) and then to 1,25-dihydroxyvitamin D, the active form of vitD]. Serum 25-(OH) D level is considered to be the best indicator of overall vitD level [17].

It is difficult to define properly normative values of vitD because the level varies according to various factors, including sun exposure, diet, and vitD supplementation. The definitions of vitD status including optimal, sufficiency, and insufficiency are not universally agreed upon; however, deficiency status of vitD is commonly accepted to be 25-(OH) D <10 ng/mL [18]–[24].

The aim of this study was to determine the prevalence of vitD deficiency in a French cohort of patients suffering from inherited ichthyosis, and to identify the factors associated with this deficiency.

## Methods

### Study design

This study was both approved by the Ethics Committee of Toulouse University Hospital and by the CNIL, the French national authority for registers. It was designed as a monocentric, prospective, observational study performed in a hospital reference center with expertise for rare skin diseases. All children (aged ≥2 years) and adults with ichthyosis, seen in our center between January 2011 and April 2012, were included. Data were collected during regular follow-up.

### Clinical data

A standardized questionnaire was used to record the following characteristics for all patients: age, sex, and form of ichthyosis. With regard to disease severity, we evaluated both erythema and scales [using visual analog scale (VAS) 0–10], and overall severity (mild, moderate, severe, or very severe). This was evaluated first by one of the two investigators and then systematically reviewed by the second using photography. Phototype, sun exposure habits, season of vitD measurement, systemic and topical treatment, intake of vitD supplements, and medical history of fractures were also recorded.

### Biological and radiological data

VitD status was defined as shown in Table 1. Serum concentration of 25-(OH) D and parathyroid hormone (PTH) were measured by chemiluminescent immunoassay. Coefficients of variation of the method were ≤7.5% and ≤4.5% for the intra-assay variation, and ≤13.6% and ≤6.5% for the inter-assay variation, for 25-(OH) D and PTH, respectively. For patients with vitD deficiency, we also investigated serum calcium, serum phosphorus, and bone mineral density (BMD) [25]–[27].

**Table 1:** **Definitions of vitamin D status**[18]–[24]

**VitD status**	**25(OH)D status(ng/ml)**
Optimal	x ≥ 30
Sufficiency	20 ≤ x < 30
Insufficiency	10 ≤ x < 20
Deficiency	x < 10

### Statistical analysis

Descriptive attributes were recorded to characterize the population. A linear correlation (Pearson coefficient r) was conducted between 25-(OH) D and PTH status. To identify factors associated with vitD deficiency, comparisons between two groups [25-(OH) D <10 ng/mL vs. ≥10 ng/mL] were conducted by univariate and multivariate logistic regression. The model included sex, age, phototype, intensity of erythema and scales, overall severity of ichthyosis, treatment with acitretin, season of vitD measurement, and sun exposure habits. All these potential predictors of vitD deficiency were first assessed individually. The odds ratio (OR) significance was determined by the Wald χ^2^ test. Predictors with p < 0.10 were subsequently assessed using multivariate analysis with a backward stepwise selection procedure. Possible interactions and multi-colinearity were examined. Whenever two or more potential risk factors were highly correlated, the predictor that was known as being more clinically significant was selected for entry. Tests were two-sided and p values <0.05 were considered significant. All statistical analyses were performed using Medcalc version 11.5.1.

## Results

### Characteristics of the study population

A total of 53 patients were included and their characteristics are presented in Table 2. The study population was young (mean age <30 years, and more than one-third were children). There was a majority of females. With regard to the clinical form of ichthyosis, half of the patients had autosomal recessive congenital ichthyosis (ARCI) and ~25% had common forms. Nearly one-third of the patients had dark skin (phototype IV–VI). Nearly half of the population declared that they strictly avoided sun exposure. There was no past medical history of bone fracture. Only eight patients had been treated by acitretin or were still receiving treatment. The pediatric population received usual systematic supplementation with vitD; however, adults did not receive any supplements. No other treatment influencing vitD metabolism (including phototherapy) was noted.

**Table 2:** **Patient characteristics** (**entire study population and patients with vitD deficiency**)

	**Total study population(n**:**53)**	**Patients with vitamin D deficiency(n:15)**
Age		
- Mean age (years) (±SD)	28 (±20)	25 (±13)
- Children (less than15 years and 3 months)/adults : n (%)	19 (36)/34 (64)	5 (33)/10 (67)
Gender		
- Female/Male : n (%)	35 (66)/18 (34)	9 (60)/6 (40)
Form of ichthyosis		
- Non syndromic ichthyosis : n (%)	47 (88.7)	13 (86.8)
- Common ichthyoses	12 (22.6)	0 (0)
- Ichthyosis vulgaris	6 (11.3)	0 (0)
- Recessive X-linked ichthyosis	6 (11.3)	0 (0)
- Autosomal recessive congenital ichthyosis	29 (54.7)	11 (73.4)
- Lamellar ichthyosis	16 (30.2)	9 (60)
- Congenital ichthyosiform erythroderma	8 (15.1)	1 (6.7)
- Bathing suit ichthyosis	4 (7.5)	1 (6.7)
- Self-healing collodion baby	1 (1.9)	0 (0)
- Keratinopathic ichthyosis (epidermolytic ichthyosis)	3 (5.7)	1 (6.7)
- Other forms	3 (5.7)	1 (6.7)
- Erythrokeratodermia variabilis	1 (1.9)	0 (0)
- Congenital reticular ichthyosiform erythroderma	1 (1.9)	1 (6.7)
- Keratosis linearis - ichthyosis congenital – keratoderma	1 (1.9)	0 (0)
- Syndromic ichthyosis : n (%)	6 (11.3)	2 (13.2)
- Netherton syndrome	3 (5.7)	0 (0)
- KID syndrome	2 (3.7)	2 (13.2)
- Neutral lipid storage disease	1 (1.9)	0 (0)
Skin features		
- Erythema (VAS) mean ± SD [range]	2.20 ± 2.5 [0 – 8.5]	2.57 ± 2.5 [0 – 8.5]
- Scaling (VAS) mean ± SD) [range]	4.25 ± 2.2 [1.5 – 9]	4.80 ± 2.6 [1.5 – 9]
Overall severity		
- Mild to moderate/Severe to very severe : n (%)	38 (72)/15 (28)	7 (47)/8 (53)
Phototype		
- I to III/IV to VI : n (%)	39 (74)/14 (26)	8 (53)/7 (47)
Sun exposure habits		
- Avoiding strictly sun exposure : n (%)	21 (45)	6 (46)
Number of patients with missing data	6	2
Season of vitamin D measurement		
- Winter – spring/Summer – autumn : n (%)	30 (57)/23 (43)	13 (87)/2 (13)
Treatments		
- Systemic therapy (acitretin) : n (%)	8 (15)	4 (27)

### Biological investigations

The mean (+SD) serum level of 25-(OH) D was 18.24 ± 10 ng/mL. In 47 of the 53 patients (88.7%), serum 25-(OH) D was below the optimal status of 30 ng/mL: 18 patients (34%) had vitD sufficiency, 14 (26.4%) had vitD insufficiency, and 15 (28.3%) had vitD deficiency. Serum PTH was abnormally high for 10% of the patients with vitD sufficiency or insufficiency, and for 42% of the patients with vitD deficiency. A negative linear correlation was statistically found between 25-(OH) D and PTH levels (r = −0.44; p = 0.003).

### Characteristics of the population with vitD deficiency

The clinical characteristics of the 15 patients with vitD deficiency are detailed in Table 2. There was no common ichthyosis in this group and >70% had ARCI. Fifty-three percent had severe or very severe disease. Nearly half had dark skin (phototype IV–VI). Serum calcium and phosphorus levels were normal for all 15 patients. BMD was investigated for 11 of these 15 patients: six of them had osteopenia, two of whom were on acitretin (Table 3).

**Table 3:** BMD measurement in patients with vitD deficiency

**Patient no**	**Age(years)**	**BMD score at lumbar spine(SD)**	**BMD score at femoral neck(SD)**	**Acitretin therapy(yes/no)**
**1**	**10**	−**1.5**	**ND**	**yes**
**2**	**13**	−**1**	**ND**	**no**
3	13	−0.8	ND	no
**4**	**24**	−**1.3**	−**0.3**	**yes**
5	23	−0.3	0.5	no
6	52	1	−0.9	no
**7**	**27**	−**1.2**	−**0.2**	**no**
**8**	**40**	−**1.7**	−**0.3**	**no**
**9**	**24**	−**1.4**	−**0.2**	**no**
10	36	−0.8	0.2	no
11	30	−0.4	0.2	no

BMD measurements were determined using dual energy X-ray absorption scanning at two standard sites: lumbar spine (L2–L4) and femoral neck (dual femur). BMD values are expressed as T score for adults, and Z score for children, as the number of the SD.

Lines in bold reflect the patients with osteopenia.

### Factors associated with vitD deficiency

Using univariate analysis (Table 4), we identified four factors that were predictors of vitD deficiency: season of vitD measurement (winter and spring) with an OR of 8.64 [95% confidence interval (CI): 1.59–46.81], p = 0.01; severity of ichthyosis (severe/very severe): 8 (1.79–35.75), p < 0.01; intensity of scales (VAS >5/10): 5.6 (1.31–24), p = 0.02; and phototype (IV–VI): 4.6 (1.11–19.14), p = 0.04. In contrast, no statistically significant association was found with sex, age, intensity of erythema, sun exposure behavior, and acitretin therapy. After adjustment, in multivariate analysis, only three factors were identified as independent risk factors: season of vitD measurement [OR (95% CI): 21.87 (1.48–323.64), p = 0.03]; severity of ichthyosis (severe/very severe) [OR (95% CI): 14.61 (1.52–140.56), p = 0.02); and phototype (IV–VI) [OR (95% CI): 10.94 (1.09–110.48), p = 0.04].

**Table 4:** **Determination of the factors specifically related to vitD deficiency** (**univariate regression analysis procedures**)

	**VitD deficiency**	
	Odds ratio (CI_95%_)	p-value
Gender (male)	1.64 [0.38 – 7.13]	0.51
Age (≥25 years)	1.6 [0.41 – 6.19]	0.5
**Phototype(IV to VI)**	**4.6[1.11–19.14]**	**0.04**
Erythema (VAS ≥ 5/10)	0.8 [0.14 – 4.66]	0.8
**Scaling(VAS** ≥ **5/10)**	**5.6[1.31–24]**	**0.02**
**Ichthyosis global severity(severe-very severe)**	**8[1.79 –35.75]**	<**0.01**
Systemic therapy (acitretin)	3.25 [0.66 – 16.04]	0.15
**Season(winter-spring)**	**8.64[1.59–46.81]**	**0.01**
Sun behavior (Sun avoidance)	1.29 [0.34 – 4.86]	0.71

Lines in bold reflect the four factors that were predictors of vitD deficiency.

## Discussion

This is believed to be the first study to evaluate the prevalence of nonoptimal vitD status in a European population with inherited ichthyoses. More than 80% did not have an optimum status of vitD, with nearly one-third having vitD deficiency. High grades of ichthyosis severity, dark skin, and winter/spring seasons were identified as independent risk factors for vitD deficiency.

Our study had several limitations. One limitation was the small number of patients included in the cohort owing to the rarity of the disease (28). Another limitation of the study was the inclusion of patients seen in an expert hospital reference center for ichthyosis where the most severe forms are seen (28% of severe or very severe forms vs 23.5% in a recent French epidemiological study [28]). Disease severity was identified as a risk factor; therefore, the prevalence in the entire French ichthyosis population (including mildest clinical forms not seen in hospital) could be slightly lower. The absence of a control group was another limitation of the study. Nevertheless, the prevalence of abnormally low vitD seems to be higher in ichthyosis than in the general healthy population. In Boston, USA, Mitchell *et al*. reported that 64% of healthy volunteers aged 18–50 years had 25 (OH) D ≤30 ng/mL [29]. In France, Vernay *et al*. reported that 80.1% of healthy volunteers aged 18–74 years had 25 (OH) D <30 ng/mL and only 4.8% had 25(OH) D <10 ng/mL [30].

Two other studies have described vitD status in ichthyosis. Ingen-Housz-Oro *et al*. reported a series of nine adults (6 Europeans and 3 Africans) living in Paris (France) and suffering from severe congenital ichthyosis [31]. All had abnormally low vitD status (25 (OH) D <30 ng/mL) and three had vitD deficiency [25 (OH) D <10 ng/mL]. The series by Chouhan *et al*. studied 45 Indian children and adolescents suffering from severe or moderate ichthyosiform erythroderma (including ichthyosis but also psoriasis and pityriasis rubra pilaris) [32]. In that series, 97% had 25-(OH) D <20 ng/mL compared with 71% of the controls. This high percentage could be explained by the presence of risk factors (all patients had dark skin and severe disease). In the same study, PTH level was significantly higher in the disease group than in the controls. As long as we find a negative linear correlation between 25-(OH) D and PTH levels, we can suppose that secondary hyperparathyroidism is linked to vitD deficiency. Nevertheless, this is in contrast with the results of the series by Milstone *et al*. who demonstrated that patients suffering from ichthyosis associated with secondary hyperparathyroidism did not have any vitD deficiency [33].

Similarly to ichthyosis, abnormal vitD has been reported in association with other skin diseases characterized by abnormal scaling. Gisondi *et al*. recently demonstrated that 58% of patients with psoriasis had vitD levels <20 ng/mL compared with 30% of the control group [34].

With regard to the three identified independent risk factors, two of these were expected: season of vitD measurement and phototype. With regard to the season, the weather is cloudy and cold in France during the winter season, days are short, and people are usually dressed in sweaters and long trousers. This limits the penetration of UV B photons into the skin and therefore increases the risk of vitD deficiency. This risk goes down in the fall and is lowest in the summer. The spring is a good time to measure vitD status because it gives an assessment of the lowest level [35]. Concerning the phototype, melanin could act as a filter and alter the synthesis of vitD in the skin by absorbing UV photons in competition with 7-dehydrocholesterol. It is therefore important to point out that patients with ichthyosis are susceptible to vitD deficiency, in part for the same reasons (season and dark skin), as other individuals who do not have ichthyosis.

The third independent identified risk factor was disease severity. This factor is specific to the disease. The mechanism underlying the link between vitD deficiency and the severity of the disease remains unclear but we can make some hypotheses. The severity of the disease may be partly related to the severity of scales (which was identified in univariate but not in multivariate analysis and probably integrated in/erased by the factor ichthyosis severity in multivariate analysis). The presence of scales increases the thickness of the skin and probably reduces UV B penetration in the skin. The influence of scales may also suggest that the intrinsic barrier defect of ichthyosis could disturb previtamin D synthesis in the skin. Further pathophysiological study should be performed to explore this hypothesis.

One important additional point that needs to be discussed is the impact of sun avoidance on vitD level in ichthyosis patients. In our study, no significant association was found between sun avoidance and vitD level. Nevertheless, it is known that because ichthyosis is a stigmatizing condition, patients usually hide their skin with clothes, and are therefore probably less exposed to the sun [2]. During the summer, they also avoid the warmth of the sun because it increases cutaneous pain and pruritus. Therefore, their exposed skin surface area may be less than that in the general population, reducing the chance for UV B photons to allow vitD synthesis in their skin. We can hypothesize that the impact of this sun avoidance is confounded with the risk factor season. This could be clarified in a future study restrictively performed during sunny seasons, and using an appropriate questionnaire (i.e. time spent outdoors, use of sunscreen, and exposed skin surface area).

Based on our results, we recommend systematic screening of 25-(OH) D in ichthyosis patients and adequate supplementation if the status is not optimal [36]. Nevertheless, the real benefit of vitD status determination and supplementation was recently challenged by Bolland *et al*. [37]. The author found that vitD supplementation did not reduce the skeletal and nonskeletal outcomes. However, this meta-analysis was performed in the general population and therefore cannot be extended to high-risk populations such as patients with ichthyosis.

## Conclusions

Clinicians should be aware of the risk of vitD deficiency in the management of patients with ichthyosis, especially in winter and spring, and in those with dark skin or severe disease. The modalities of supplementation and follow-up of such patients could be better defined in further studies.

## Abbreviations

25-(OH) D: 25-hydroxyvitamin D

ARCI: Autosomal recessive congenital ichthyosis

BMD: Bone mineral density

CI: Confidence interval

OR: Odds ratio

PTH: Parathyroid hormone

SD: Standard deviation

VAS: Visual analog scale

vitD: vitamin D

UVB: Ultraviolet B

## Competing interests

The authors declare that they have no competing interests.

## Authors’ contributions

FF participated in design and coordination of the study, contributed to collecting data, evaluated disease severity, performed revision and interpretation of all the clinical data, participated in statistical analyses, and wrote the manuscript. ID participated in design and coordination of the study, contributed to the interpretation of clinical data, performed the statistical analyses, and co-wrote the manuscript. JS contributed towards interpreting the clinical data and revising the manuscript. IG contributed towards interpreting the clinical data and revising the manuscript. LR contributed to collecting and revising data. KE contributed to revising the manuscript. JMH designed the study and co-coordinated the study, systematically reviewed disease severity using photography, contributed to the acquisition and interpretation of data, and co-wrote the manuscript. All authors read and approved the final manuscript.
